# Disparate Central and Peripheral Effects of Circulating IGF-1 Deficiency on Tissue Mitochondrial Function

**DOI:** 10.1007/s12035-019-01821-4

**Published:** 2019-11-15

**Authors:** Gavin Pharaoh, Daniel Owen, Alexander Yeganeh, Pavithra Premkumar, Julie Farley, Shylesh Bhaskaran, Nicole Ashpole, Michael Kinter, Holly Van Remmen, Sreemathi Logan

**Affiliations:** 1 Department of Physiology, University of Oklahoma Health Sciences Center, Oklahoma City, OK USA; 2 Aging & Metabolism Research Program, Oklahoma Medical Research Foundation, Oklahoma City, OK USA; 3 Oklahoma Center for Geroscience, University of Oklahoma Health Sciences Center, Oklahoma City, OK USA; 4 Oklahoma Center for Neuroscience, University of Oklahoma Health Sciences Center, Oklahoma City, OK USA; 5 Department of Biomolecular Sciences, University of Mississippi, Oxford, MS USA; 6 Department of Rehabilitation Sciences, College of Allied Health, University of Oklahoma Health Sciences Center, Oklahoma City, OK USA

**Keywords:** IGF-1, Mitochondria, ROS, Cognitive function, Learning and memory, Oxidative stress

## Abstract

Age-related decline in circulating levels of insulin-like growth factor (IGF)-1 is associated with reduced cognitive function, neuronal aging, and neurodegeneration. Decreased mitochondrial function along with increased reactive oxygen species (ROS) and accumulation of damaged macromolecules are hallmarks of cellular aging. Based on numerous studies indicating pleiotropic effects of IGF-1 during aging, we compared the central and peripheral effects of circulating IGF-1 deficiency on tissue mitochondrial function using an inducible liver IGF-1 knockout (LID). Circulating levels of IGF-1 (~ 75%) were depleted in adult male *Igf1*^*f/f*^ mice via AAV-mediated knockdown of hepatic IGF-1 at 5 months of age. Cognitive function was evaluated at 18 months using the radial arm water maze and glucose and insulin tolerance assessed. Mitochondrial function was analyzed in hippocampus, muscle, and visceral fat tissues using high-resolution respirometry O2K as well as redox status and oxidative stress in the cortex. Peripherally, IGF-1 deficiency did not significantly impact muscle mass or mitochondrial function. Aged LID mice were insulin resistant and exhibited ~ 60% less adipose tissue but increased fat mitochondrial respiration (20%). The effects on fat metabolism were attributed to increases in growth hormone. Centrally, IGF-1 deficiency impaired hippocampal-dependent spatial acquisition as well as reversal learning in male mice. Hippocampal mitochondrial OXPHOS coupling efficiency and cortex ATP levels (~ 50%) were decreased and hippocampal oxidative stress (protein carbonylation and F_2_-isoprostanes) was increased. These data suggest that IGF-1 is critical for regulating mitochondrial function, redox status, and spatial learning in the central nervous system but has limited impact on peripheral (liver and muscle) metabolism with age. Therefore, IGF-1 deficiency with age may increase sensitivity to damage in the brain and propensity for cognitive deficits. Targeting mitochondrial function in the brain may be an avenue for therapy of age-related impairment of cognitive function. Regulation of mitochondrial function and redox status by IGF-1 is essential to maintain brain function and coordinate hippocampal-dependent spatial learning. While a decline in IGF-1 in the periphery may be beneficial to avert cancer progression, diminished central IGF-1 signaling may mediate, in part, age-related cognitive dysfunction and cognitive pathologies potentially by decreasing mitochondrial function.

**Electronic supplementary material:**

The online version of this article (10.1007/s12035-019-01821-4) contains supplementary material, which is available to authorized users.

## Introduction

Aging is associated with reductions in mitochondrially derived energy production [1], global increases in oxidative load (reviewed by [2, 3]), and a concomitant decline in correlates of cognitive function, specifically neuronal and astroglial function [4–6]. Dysregulation of mitochondrial function, including diminished ATP synthesis, excessive free radical production, and apoptosis, is part of the etiology of many age-related human diseases, including neurodegenerative disorders such as Alzheimer’s disease [7, 8].

Insulin-like growth factor-1 (IGF-1) is a potent neurotrophic factor that is abundantly expressed in the central nervous system during embryonic development and peaks during puberty, a period of rapid growth and development [9]. Subsequently, later in life, IGF-1 levels decline substantially with age [10] both centrally (~ 30% decline) and peripherally (~ 70% decline) [11]. IGF-1 is produced and secreted into the bloodstream primarily by the liver in response to growth hormone (GH) secreted by the anterior pituitary. IGF-1 produced by the liver is transported by IGF-1 binding proteins (IGFBP) and crosses the blood-brain barrier. In addition to circulating IGF-1, this peptide is also produced locally in the brain and other tissues and has autocrine/paracrine actions. IGF-1 ligand binding to its cognate receptor, insulin-like growth factor receptor (IGFR), induces conformational changes that initiate receptor autophosphorylation of the intracellular kinase domains [12] that activate downstream signaling cascades including the PI3K/AKT and MAPK pathways that drive cell survival and growth [13].

The role of IGF-1 in cognitive and systemic aging is controversial due, in part, to the pleiotropic effects of IGF-1 [14–17]. Models of early life IGF-1 deficiency by disruption of either GH/IGF-1 axis or DAF2/insulin signaling have reported increases in lifespan in nematodes, flies, and mice [18, 19]. Conversely, reduced IGF-1 has been associated with oxidative stress [20, 21] and increased risk of cardiovascular disease [22, 23], stroke [24, 25], and cognitive decline [11, 14, 26, 27]. While reduction in IGF-1 peripherally protects against the onset and progression of specific types of cancer [14] and modestly increases lifespan in a sex-dependent manner, deficiency of IGF-1 availability in the brain by ectopic expression of IGF-1 binding protein (IGFBP-1) has been shown to reduce astrocytic response to injury [28]. IGF-1 has also been shown to protect neurons against oxidative stress via modulation of astrocytic responses [29, 30]. IGF-1 signaling regulates ATP production in aging mice [31] and has recently been shown to affect mitochondrial dynamics in astroglial cells [32]. However, the role of IGF-1 in regulating mitochondrial function centrally and peripherally is not well understood, and the mechanism by which IGF-1 mediates cognitive performance and preserves peripheral tissue function remains controversial.

In this study, we investigated the central and peripheral effects of circulating IGF-1 deficiency on tissue mitochondrial function. We show that IGF-1 deficiency does not significantly impact muscle mitochondrial function, although fat metabolism was increased. The latter could be attributed to increases in growth hormone in this model that has been shown to increase fat metabolism. More importantly, we show that circulating IGF-1 deficiency significantly impaired hippocampal-dependent spatial acquisition as well as reversal learning in male mice. These behavioral data correlated with decreased brain ATP levels and hippocampal mitochondrial OXPHOS coupling efficiency. Furthermore, IGF-1 knockdown increased hippocampal oxidative stress and stress-related gene expression. These data suggest that IGF-1 is critical for mitochondrial function in the central nervous system and coordinates spatial learning. IGF-1 deficiency with age may increase sensitivity to damage in the brain and propensity for cognitive deficits and targeting mitochondrial function in the brain may be an avenue for therapy for age-related impairment of cognitive function.

## Materials and Methods

### Animals

All procedures were approved by and followed the guidelines of the Institutional Animal Care and Use Committee of OUHSC. Only male mice were used in this study. Young (4–6 months) C57Bl/6 and *Igf1*^*f/f*^ (B6.129(FVB)-Igf1tm1Dlr/J) mice were obtained from Jackson laboratories. Mice were housed (3–4 per cage) in Allentown XJ cages with Anderson’s Enrich-o-cob bedding (Maumee, OH). C57Bl/6 mice were bred in house to generate experimental cohorts. These mice were housed in the Rodent Barrier Facility (RBF) at OUHSC, which is a specific pathogen-free (including helicobacter and parvovirus) facility. Mice were bred on a 14-h light/10-h dark cycle and weaned mice were maintained in a 12-h light/12-h dark cycle at 21 °C and were given access to standard irradiated bacteria-free rodent chow (5053 Pico Lab, Purina Mills, Richmond, IN) and reverse osmosis filtered water ad libitum.

### Liver IGF-1-Deficient (LID) Mice

**Fig. 1:**
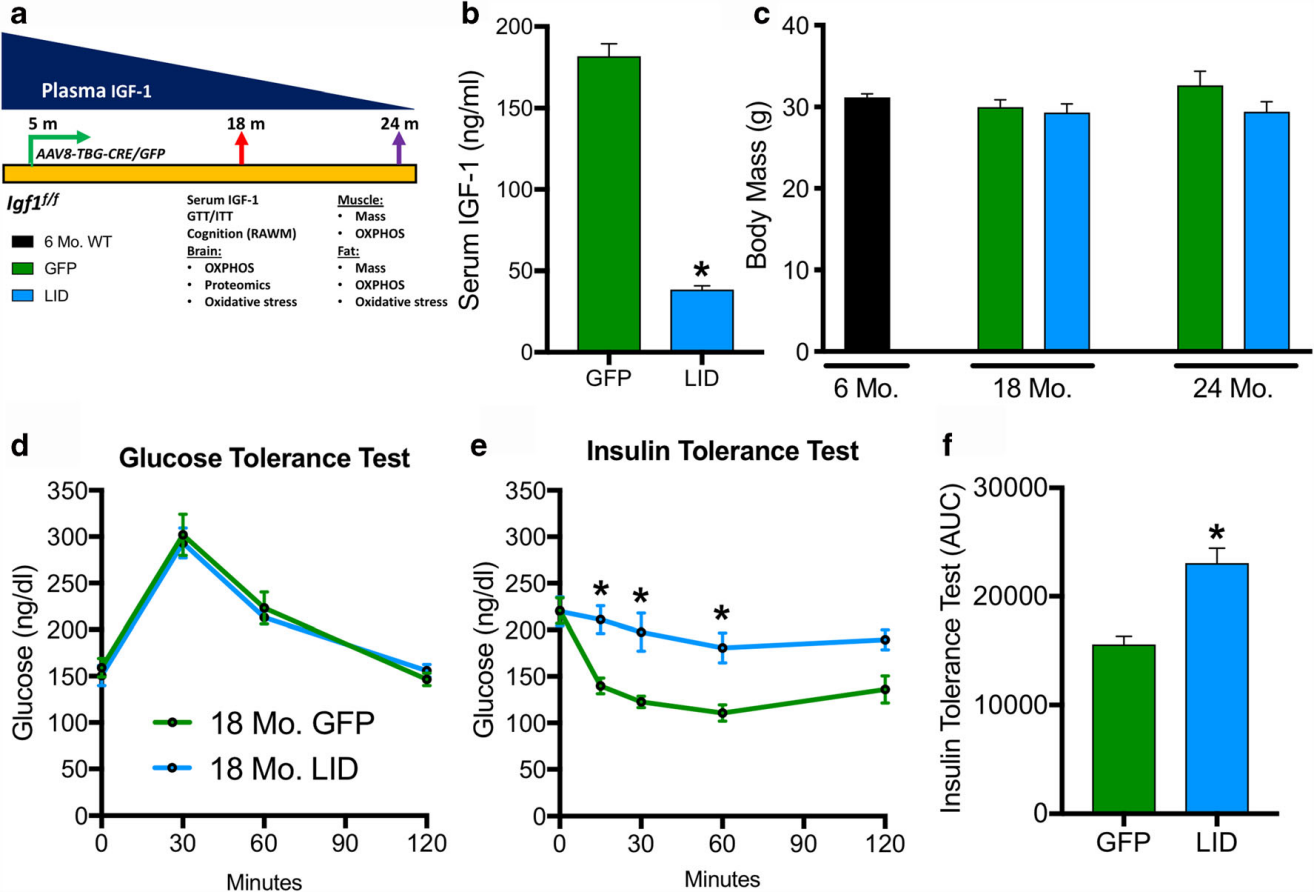
Liver IGF-1 deficiency (LID) increases insulin resistance. **a** Experimental timeline of circulating IGF-1 deficiency induced by liver-specific AAV8-TBG-Cre (LID) or AAV8-TBG GFP (GFP) illustrating the timepoints for induction and functional analyses. **b** Circulating IGF-1 in the serum is significantly reduced in the LID (*n* = 12) compared with GFP (*n* = 6) mice at 18 months of age. **c** Body mass was not different between GFP (green) and LID (blue) mice at 18 months and 24 months and was comparable with young WT (black) mice (6-month reference group; *n* = 6). **d** Glucose tolerance test (GTT) and **e** insulin tolerance test (ITT) in 18-month GFP and LID mice and **f** area under the curve (AUC) for insulin tolerance test (*n* = 6–8). GTT/ITT were analyzed by two-way ANOVA with Sidak’s post hoc test (*p < 0.05). Serum IGF-1 and ITT AUC were compared with two-tailed student’s *t* test (*p < 0.05). Data are represented as the mean ± SEM. Radial arm water maze (RAWM); oxidative phosphorylation (OXPHOS)

*Igf1*^*f/f*^ mice (C57Bl/6J background) were injected retro-orbitally with *AAV8-TBG-GFP* (control mice) or *AAV8-TBG-GFP-CRE* (LID mice) at 4–5 months of age as previously described [14]. Mice were housed and evaluated at specific timepoints (18 months and 23–24 months chronological age; Fig. 1a) for behavioral and molecular endpoints.

### IGF-1 ELISA

Whole blood was isolated from the submandibular vein and centrifuged (2500×*g* for 20 min at 4 °C), sera removed, and frozen at – 80 °C until further analysis. IGF-1 concentrations were quantified using Mouse IGF-1 Quantikine ELISA kit (R&D Systems, Minneapolis, MN) as described previously [14]. All analytes were measured in duplicate following the manufacturers’ recommendations.

### Glucose and Insulin Tolerance Test

Glucose tolerance test (GTT) was performed on control (*n* = 6) and LID (*n* = 8) mice at 1 year post knockdown (15-month chronological age). Fasted mice (16 h) were orally administered 1.5 g/kg of D-glucose (Sigma). Prior to and after glucose administration, blood glucose levels were recorded from tail vein at 0, 30, 60, 120, and 150 min. Mice were returned to their home cages and allowed to recover for 1 week.

Subsequently, an insulin tolerance test (ITT) was performed on the same mice. Mice were fasted for 5 h during the day, then injected with 0.75 units/kg body weight of human insulin (Sigma) and blood glucose measured as before at 0, 15, 30, 60, and 120 min from tail vein. Area under the curve (AUC) was determined for each mouse using GraphPad Prism version 7.0b for Mac OSX.

### Energy Charge Measurements and Glutathione Redox Status

Mice were rapidly decapitated, their brain exposed within the skull, and whole heads were flash frozen in liquid nitrogen (≤ 15 s) to avoid degradation of ATP and other metabolites. Control and *Igf1* knockout (LID) brains and eWAT (*n* = 6/group) were then scraped under liquid nitrogen and processed. Samples were extracted with 300 μl of 300 mM potassium hydroxide. The resulting extract was centrifuged at 20,000×*g* for 15 min, then filtered and resolved by high-performance liquid chromatography (Shimadzu LC-20A High Precision Binary Gradient HPLC system; Shimadzu, Kyoto, Japan). A UV/VIS diode array spectrometer was used to detect AMP, ADP, and ATP at 254 nm and quantified on the basis of the integrated area of standards [33]. Energy charge was calculated by using the equation: ([ATP] + 0.5[ADP])/ ([ATP] + [ADP] + [AMP]).

The intracellular ratio of NAD^+^ to NADH in cell lysates was measured in cortex by using an enzyme recycling reaction to quantitate NADH absorbance in accordance with the instructions of the manufacturer (BioVision, Inc., Milpitas, CA, USA) [33]. Oxidized and reduced glutathione were measured spectrophotometrically in both cortex and eWAT (Tecan US) by using an enzymatic recycling method to quantify the production of 5-thio-2-nitrobenzoic acid (TNB) generated from the reaction of reduced glutathione (GSH) with 5′-5′ dithio-*bis*-2 (nitrobenzoic acid) (DTNB) in accordance with the instructions of the manufacturer (Cayman Chemicals Company).

### Hippocampus Mitochondrial Isolation and Muscle and Fat Biopsy preparation

Mice (18 months) were rapidly decapitated and hippocampi were dissected from the brain into mitochondria isolation buffer-A (10 mM HEPES, 250 mM D-Mannitol, 75 mM sucrose, 100 μM EDTA, 500 μM EGTA, 0.1% BSA, and protease inhibitor cocktail (Roche)). Two hippocampi from one mouse were pooled for *n* = 1. Mitochondria were isolated as previously described [32]. Hippocampi were homogenized with 30 strokes with a Dounce homogenizer. The homogenate was spun at 1500×*g* for 10 min to pellet nuclei. The resulting supernatant was spun at 20,000×*g* for 20 min and the resulting mitochondrial pellet was resuspended in assay buffer [125 mM KCl, 10 mM HEPES, 5 mM MgCl2, 2 mM K2HPO4, pH 7.44] for analysis by high-resolution respirometry (HRR). Protein concentration was determined by the Bio-Rad DC assay (Bio-Rad, Hercules, CA).

Muscle fiber and fat biopsies were isolated from young (6 months) and aged control and LID mice (24 months). Red gastrocnemius muscle fibers (~ 3–5 mg) were separated in buffer X containing (in mM) 7.23 K_2_EGTA, 2.77 CaK_2_EGTA, 20 imidazole, 0.5 DTT, 20 taurine, 5.7 ATP, 14.3 PCr, 6.56 MgCl_2_–6H_2_O, and 50 K-MES (pH 7.1). The fibers were permeabilized in buffer X with 30 μg/ml saponin, washed 3 times for 5 min in buffer Z containing (in mM) 105 K-MES, 30 KCl, 10 K_2_HPO_4_, 5 MgCl_2_–6H_2_O, 0.5 mg/ml bovine serum albumin (BSA), and 0.1 EGTA (pH 7.1), then loaded in the O2K. eWAT (~ 50 mg) was sliced into ~ 1 mm^3^ pieces and loaded directly into the O2K. Mitochondria (100 μg) isolated from hippocampi were loaded directly into the O2K.

### High-Resolution Respirometry and Fluorometry

We simultaneously probed oxygen consumption rate (OCR) and hydroperoxide production rate in permeabilized gastrocnemius muscle fibers, epididymal white adipose tissue (eWAT) biopsies, and isolated hippocampus mitochondria using HRR (OROBOROS Instruments, Innsbruck, Austria) and the O2K-Fluo LED2-Module Fluorescence-Sensor Green with the fluorogenic probe Amplex UltraRed as previously described [34, 35]. Hydroperoxide groups, including hydrogen peroxide and lipid hydroperoxides, interact with Amplex UltraRed in a reaction catalyzed by horseradish peroxide to form the fluorescent compound resorufin [36]. The rate of increase in fluorescence corresponds to the rate of hydroperoxide production. The fluorescent signal was converted to nanomolar H_2_O_2_ via a standard curve established on each day of experiments. Background resorufin production was subtracted from each measurement. Measurements were performed in buffer Z at 37 °C containing 10 μM Amplex® UltraRed (Molecular Probes, Eugene, OR), 1 U/ml horseradish peroxidase (HRP), 12.5 U/ml superoxide dismutase (SOD), and blebbistatin (25 μM).

We measured both OCR and hydroperoxide production rate during sequential addition of sample, complex I substrates (10 mM glutamate and 2 mM malate), 2.5 mM ADP, complex II substrate (10 mM succinate), complex I inhibitor (1 μM rotenone), complex III inhibitor (1 μM Antimycin A), and complex IV substrates (5 mM ascorbate and 0.5 mM TMPD). We normalized the OCR using non-mitochondrial respiration after addition of antimycin A. LEAK state respiration is the oxygen consumed in the presence of substrates but absence of ADP to maintain the mitochondrial membrane potential against protons that leak through the inner membrane. Oxidative phosphorylation (OXPHOS) capacity is the maximum ability to consume oxygen with supraphysiological complex I and II substrates and ADP [37]. Data for OCR and hydroperoxide production rate were normalized by wet weight for muscle fibers, wet weight for fat biopsies, and microgram of protein for isolated hippocampus mitochondria determined by Bradford protein assay.

### Targeted Quantitative Proteomics

Frozen mouse brain hippocampi were processed for GFP and LID mice (*n* = 6). The tissues were homogenized in RIPA buffer and a volume equivalent to 100 μg protein was taken for analysis as previously described [38]. The samples were analyzed on a TSQ Vantage triple quadrupole mass spectrometry system with HPLC Eksigent splitless nanoflow. A BSA internal standard was added for quantification, and the mass spectrometer was operated in selected reaction monitoring mode to analyze two peptides per protein. Data were analyzed using the program SkyLine, and the response for each protein was calculated as the geometric mean of the two peptide area normalized to the response for the BSA standard. The principal component analysis (PCA) plot was generated using ClustVis with default settings (Row scaling = unit variance scaling, PCA method = SVD with imputation, clustering distance for rows = correlation, clustering method for rows = average, tree ordering for rows = tightest cluster first) [39]. Additional methodological details are available as supplemental methods.

### F_2_-Isoprostane Quantification

Levels of F_2_-isoprostanes in cortex were determined by a previously described method with minor modifications [40]. Briefly, 200 mg of cortex was homogenized in 10 ml of ice-cold Folch solution (CHCl_3_:MeOH, 2:1) containing butylated hydroxytoluene (BHT). The mixture was incubated at room temperature for 30 min. Two milliliters of 0.9% NaCl (w/v) was added and mixed. The homogenate was centrifuged at 3000×*g* for 5 min at 4 °C. The aqueous layer was discarded, while the organic layer was secured and evaporated to dryness under N_2_ at 37 °C. Esterified F_2_-isoprostanes were extracted and quantified by gas chromatography-mass spectrometry using the internal standard [^2^H_4_]8-Iso-PGF_2α_, which was added to the samples at the beginning of extraction to correct yield of the extraction process. The level of F_2_-isoprostanes in cortex was expressed as nanograms of 8-Iso-PGF_2α_, per gram of mass.

### Protein Carbonylation

Cortex protein carbonyls were measured as previously described in skeletal muscle [41, 42]. Briefly, frozen cortex was homogenized in sodium phosphate buffer. The hydrazine reagent, fluorescein-5-thiosemicarbazide (FTC, Molecular Probes, Eugene, OR, USA) (1 mM), was added and incubated for 2 h at 37 °C in the dark. The excess unreacted FTC was removed and equal amounts of protein were loaded on a 12% sodium dodecyl sulfate polyacrylamide gel electrophoresis to resolve the FTC-labelled proteins. A fluorescence scan of the gel was used to measure the amount of bound FTC, and the gel was stained with Coomassie blue. The carbonyl content of the protein samples was expressed as the ratio of FTC fluorescence (carbonyls) to Coomassie blue absorption (protein concentration).

### Radial Arm Water Maze (RAWM)

Mice (young and old C57Bl/6; *n* = 10/group) were tested for spatial learning using an 8-arm radial arm water maze during the morning hours. Mice were given 8 times for 60-s trials a day for 3 days to find a hidden platform in one arm of an 8-arm radial arm water maze that is 67 cm in diameter filled with an opaque liquid. Animals were randomly placed in an arm other than the target arm. Mice were guided to the platform if they failed to find the target at the end of each 60-s trial. Animal movements in the maze were recorded by a camera and tracked using an automated tracking system (Noldus Ethovision XT 11, Wageningen, Netherlands). The number of errors (no. of entries into incorrect arms) and path length (cm; total distance travelled) to target were recorded. An error was counted when an animal had traversed 2/3 the length of the arm that did not have the platform. Mice were tested during the day (in the morning hours) for 3 days to find a hidden platform in one arm (4 times 60-s trials), following which they were returned to their home cage. One week later (day 10), the ability of the mice to remember the position of the platform was assessed with 4 times 60-s trials. The next day (day 11), the platform was moved to a new location and mice were tested again with 8 times for 60-s trials to relearn a new position (extinction).

### Statistical Analyses

All experiments were performed in multiple independent replicates per group as described for each experiment. Statistical differences between experimental groups for RAWM experiments were analyzed using a multivariate repeated measures ANOVA followed by Dunnett’s post hoc test. Data are represented as the mean ± SEM. Significance is indicated by *p* value measurements with a *p* < 0.05 considered significant: *p < 0.05; **p < 0.01; ***p < 0.001. All other statistical methods were performed using GraphPad Prism version 7.0b for Mac OS X. Statistical significance for experiments with two groups was determined using unpaired two-tailed student’s *t* test with *p* < 0.05 considered significant. Statistical significance for experiments with three groups was determined using one-way ANOVA with Tukey’s post hoc test where *p* < 0.05 is considered significant. Linear regression was determined using GraphPad Prism. Statistical significance for protein content by mass spectrometry was assessed by one-way ANOVA with Tukey’s post hoc test and Benjamini-Hochberg correction. For the quantitative mass spectrometry, energy charge measurements and glutathione redox status, and F_2_-isoprostane quantification, the Grubb’s outlier test was used to control for outliers in the data. For data sets with statistically different variance, the variance was normalized by transforming the data sets before running tests for statistical difference.

## Results

### Liver IGF-1 Deficiency Increases Insulin Resistance

To understand the role of IGF-1 deficiency in adulthood on central and peripheral tissue metabolic functions, we knocked down liver production of IGF-1 at 4–5 months of age. AAV8-TBG-Cre or AAV8-TBG-GFP were injected in *Igf1*^*f/f*^ male mice as previously described [14]. The timeline for the experiment is outlined in Fig. 1a. Functional and biochemical endpoints were assessed at 18 months and 24 months chronological age as illustrated. Cognitive testing and further analyses on the brain were conducted at 18 months based on previous studies that showed a decline in hippocampal neurogenesis by 18 months which persisted up to 28 months that was ameliorated by IGF-1 infusion [43]. Mitochondrial functional testing on muscle and fat tissue were conducted at approximately 24 months, since previous studies on aged mice and with IGF-1 transgene expression in skeletal muscle have typically looked at 22–24-month mice [44].

Levels of IGF-1 in the serum of LID mice were significantly lower (~ 70% reduction; *p* < 0.001) than aged matched control (GFP) mice at 18 months of age (~ 1 year of knockdown; Fig. 1b). Body mass was not significantly different in either group at 18 months and 24 months, but there was a trend for lower body weight in the LID mice at 24 months (Fig. 1c). Control 6-month C57Bl/6J mice were used as a reference group for body weight comparisons. We then tested whether IGF-1 deficiency had any effect on glucose or insulin processing. No differences in glucose tolerance between the two groups (Fig. 1d) were observed; however, LID mice were more insulin resistant (*p* < 0.05) than the age matched control (GFP) mice (Fig. 1e, f). Insulin resistance may result from increased production of GH in this model [14, 45, 46] as a compensatory mechanism for reduced feedback inhibition by IGF-1.

### IGF-1 Deficiency Does Not Alter Age-Related Changes in Muscle Mass or Mitochondrial Function

**Fig. 2:**
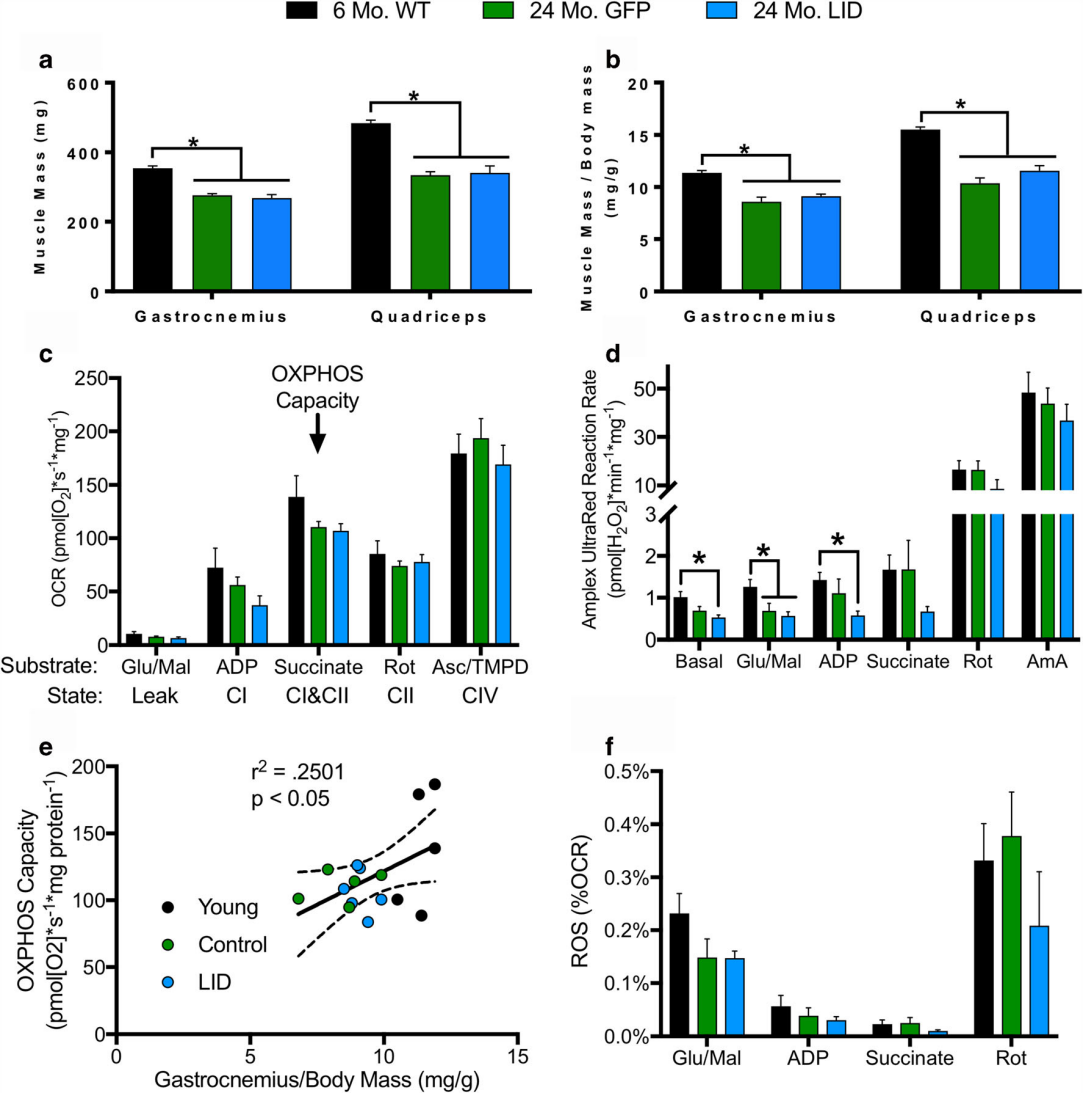
IGF-1 deficiency does not alter age-related changes in muscle mass or mitochondrial function. Decrease in **a** raw muscle mass and **b** muscle mass normalized to body mass in gastrocnemius and quadriceps femoris muscles from 24-month GFP and LID mice compared to 6-month WT (*n* = 6). Simultaneous **c** oxygen consumption rate (OCR) and **d** hydroperoxide production rate (ROS) in permeabilized red gastrocnemius fibers from 6-month WT, 24-month GFP, and 24-month LID mice measured in the O2K with Amplex UltraRed and normalized to wet fiber mass (*n* = 6). **e** Linear regression between gastrocnemius fiber OXPHOS capacity and normalized gastrocnemius mass. **f** Amplex UltraRed Reaction Rate (ROS) as a percentage of oxygen consumption rate (OCR) in gastrocnemius fibers. Statistical significance determined by ordinary one-way ANOVA with Tukey’s Multiple Comparison Test (*p < 0.05). Box plots depicted as mean ± SEM. Six-month WT (black); 24-month GFP (green); 24-month LID (blue). Glutamate (Glu); malate (Mal); adenosine diphosphate (ADP); rotenone (Rot); ascorbate (Asc); N,N,N′,N′-tetramethyl-p-phenylenediamine dihydrochloride (TMPD); antimycin A (AmA); ETC complex I (CI); ETC complex II (CII); ETC complex IV (CIV)

The GH/IGF-1 axis regulates post-natal body growth and the proportional increase in muscle mass, which is evident from the reduced body/muscle size in early life knockouts of GH [47–49] or IGF-1 [50, 51]. We therefore investigated whether circulating IGF-1 deficiency had any effects on muscle mass in aged mice. Actual body mass and normalized hind limb muscle mass declines with age in the mouse gastrocnemius and quadriceps muscles; however, we observed no differences in muscle mass caused by IGF-1 deficiency (Fig. 2a, b).

IGF-1 has been shown to be essential for mitochondrial biogenesis and turnover in cancer cells [52] and plays a protective role in regulating mitochondrial function [53–55]. We therefore examined whether circulating IGF-1 deficiency influenced muscle mitochondrial function. We simultaneously probed oxygen consumption rate (OCR) and hydroperoxide production rate in permeabilized gastrocnemius muscle fibers using the fluorogenic probe Amplex UltraRed in O2K HRR. Hydroperoxide groups, including hydrogen peroxide and lipid hydroperoxides, interact with Amplex UltraRed in a reaction catalyzed by horseradish peroxide to form the fluorescent compound resorufin [36]. The rate of increase in fluorescence corresponds to the rate of hydroperoxide production. We measured OCR and hydroperoxide production rate during sequential addition of sample, complex I substrates (glutamate and malate), ADP, complex II substrate (succinate), complex I inhibitor (rotenone), complex III inhibitor (antimycin A), and complex IV substrates (ascorbate and TMPD).

No significant difference was observed in oxygen consumption rate, though there is a non-significant decrease in OXPHOS capacity observed in both 24-month GFP and LID muscle fibers (Fig. 2c). We observed a significant decrease in hydroperoxide production rate under certain conditions in muscle fibers from 24-month GFP and LID mice, but there were no differences in hydroperoxide production capabilities following ETC inhibitors as a result of IGF-1 deficiency (Fig. 2d). Plotting OXPHOS capacity versus normalized gastrocnemius mass reveals a significant correlation between OXPHOS capacity and gastrocnemius mass (Fig. 2e). Thus, the age-related decline with OXPHOS capacity (GFP and LID mice) parallels a decline in gastrocnemius muscle mass. We previously reported no significant difference in hydroperoxide production capability in aged gastrocnemius fibers [34]. Analyzing hydroperoxide production as a percentage of oxygen consumed revealed no significant difference between groups, suggesting that decreased hydroperoxide production in 24-month GFP and LID mouse muscle fibers is a correlate of decreased OCR (Fig. 2f).

### Liver IGF-1 Deficiency Reduces Adipose Mass and Increases Lipid Peroxidation

**Fig. 3:**
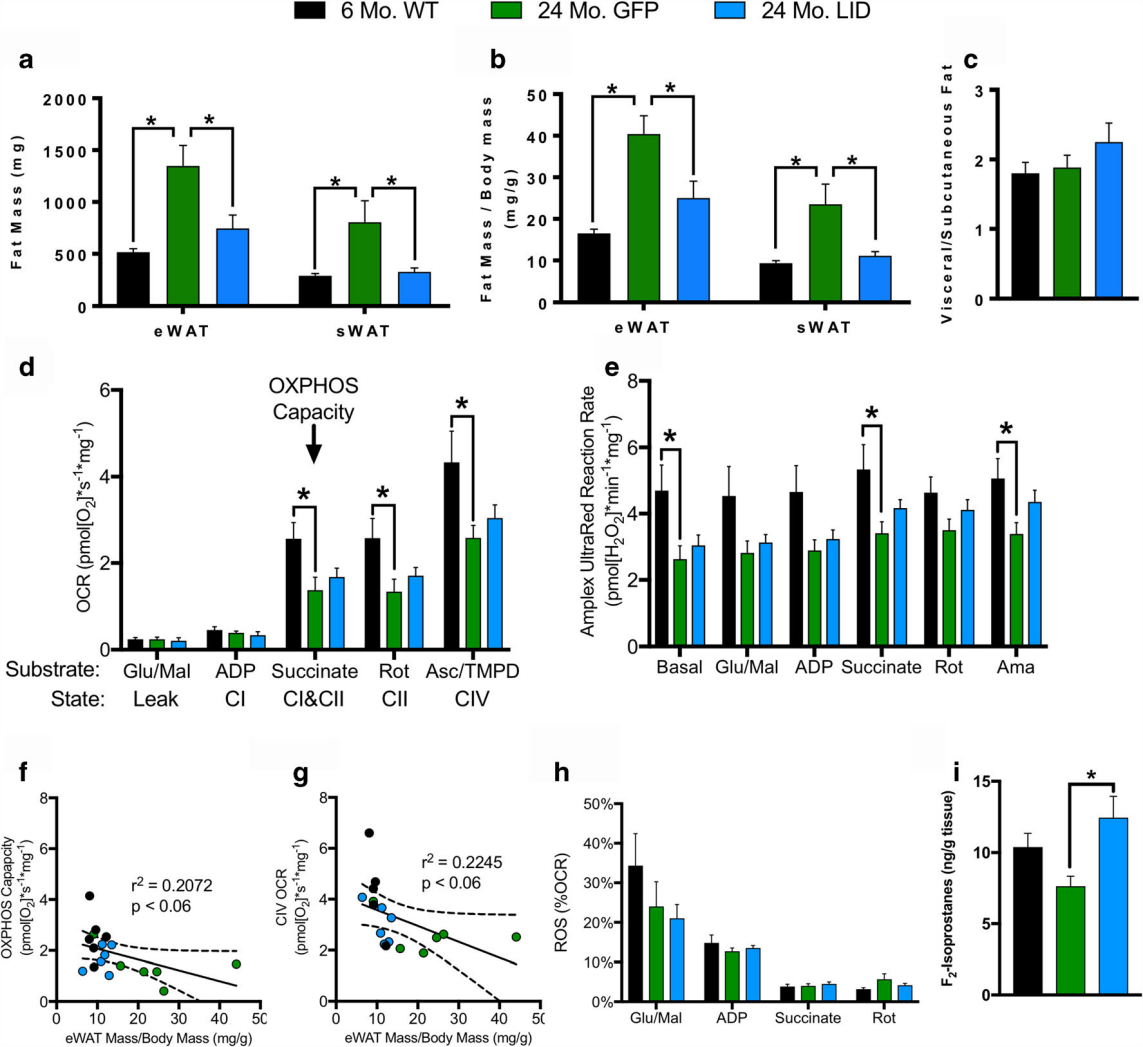
IGF-1 protects against age-related increase in adipose mass and decrease in adipose mitochondrial function. Change in **a** raw fat mass and **b** fat mass normalized to body mass in epididymal white adipose tissue (eWAT) and subcutaneous white adipose tissue (sWAT) fat pads, **c** and ratio of visceral (eWAT) to subcutaneous (sWAT) fat from 24-month GFP and LID mice compared with 6-month WT (*n* = 6). Simultaneous **d** oxygen consumption rate (OCR) and **e** hydroperoxide production rate (ROS) production in eWAT sections from 6-month WT and 24-month GFP and LID mice measured in the O2K with Amplex UltraRed and normalized to wet tissue mass (*n* = 6). Linear regression between **f** eWAT OXPHOS Capacity and normalized eWAT mass and **g** CIV OCR and normalized eWAT mass (*n* = 6). **h** eWAT Amplex UltraRed Reaction Rate (ROS) as a percentage of oxygen consumption rate (OCR) (*n* = 6). **i** Lipid hydroperoxides measured using F_2_-isoprostanes in eWAT from 6-month WT and 24-month GFP and LID mice (*n* = 6). Statistical significance determined by ordinary one-way ANOVA with Tukey’s Multiple Comparison Test (*p < 0.05). Box plots depicted as mean ± SEM. 6-month WT (black); 24-month GFP (green); 24-month LID (blue). Glutamate (Glu); malate (Mal); adenosine diphosphate (ADP); rotenone (Rot); ascorbate (Asc); N,N,N′,N′-tetramethyl-p-phenylenediamine dihydrochloride (TMPD); antimycin A (AmA); ETC complex I (CI); ETC complex II (CII); ETC complex IV (CIV)

The GH/IGF-1 axis plays an important role in lipid metabolism and homeostasis in organismal development and aging. IGF-1 stimulates preadipocyte differentiation but has a limited effect on mature adipocytes [56]. However, partial IGF-1 deficiency decreases expression of genes involved in lipid metabolism [57]. To determine the effect of reduced circulating IGF-1 on lipid metabolism, we measured both subcutaneous fat mass and epididymal fat mass and mitochondrial function. Fat mass accumulates with age in the epididymal (eWAT) and subcutaneous white adipose tissue (sWAT) fat pads expressed as absolute levels or after normalized to body mass, but mice deficient in IGF-1 were resistant to this change (Fig. 3a, b). However, we observed no change in the ratio between visceral (eWAT) to subcutaneous (sWAT) fat pads (Fig. 3c).

We simultaneously probed oxygen consumption rate (OCR) and hydroperoxide production rate in eWAT using O2K HRR (Fig. 3d, e). We observed no age-related change in LEAK state or ETC complex I-stimulated respiration; however, CI&CII (OXPHOS capacity), CII, and CIV-linked respiration were decreased with age (Fig. 3d). IGF-1-deficient animals had a modest (~ 20%) increase in respiration under these states compared with 6-month WT. We observed a trend between both decreased OXPHOS capacity and complex IV-linked respiration with increased normalized eWAT mass suggesting eWAT OCR is decreased by the age-related increase in visceral fat pad mass (Fig. 3f, g). Surprisingly, aging was associated with decreased hydroperoxide production detected by Amplex UltraRed; additionally, the ETC inhibitors rotenone and antimycin A did not increase Amplex UltraRed reaction rate (Fig. 3e). IGF-1-deficient animals have a modest (~ 10–30%) increase in Amplex UltraRed reaction rate under these conditions. Because the inhibitors rotenone and antimycin A did not increase the Amplex UltraRed reaction rate as seen in other tissues, we concluded that the source of the reactants may not originate from the ETC. Furthermore, there were no significant differences between hydroperoxide production as a percentage of OCR, suggesting that the decline in hydroperoxide production rate is caused by the decline in OCR (Fig. 3h).

We also measured the ratio of reduced (GSH) to oxidized (GSSG) glutathione and lipid peroxides as markers of oxidative stress in 6-month WT, 24-month GFP, and 24-month LID eWAT. The glutathione antioxidant system is composed of antioxidant enzymes that use the reduced form of glutathione (GSH) as a co-factor to repair oxidative damage by reducing disulfide bonds [58]. We found no difference in GSH, GSSG, or GSSG ratio between the groups (Supplemental Table 1). F_2_-isoprostanes are a reliable marker of lipid peroxidation, which is formed by free radical reaction with arachidonic acid [40]. We found a significant increase in F_2_-isoprostanes in 24-month LID eWAT compared with eWAT from aged GFP (Fig. 3i, Supplemental Table 1). Thus, circulating IGF-1 deficiency induced oxidative damage related to lipid species in eWAT irrespective of increased lipid metabolism.

### Circulating IGF-1 Deficiency Impairs Hippocampal-Dependent Spatial Learning

**Fig. 4:**
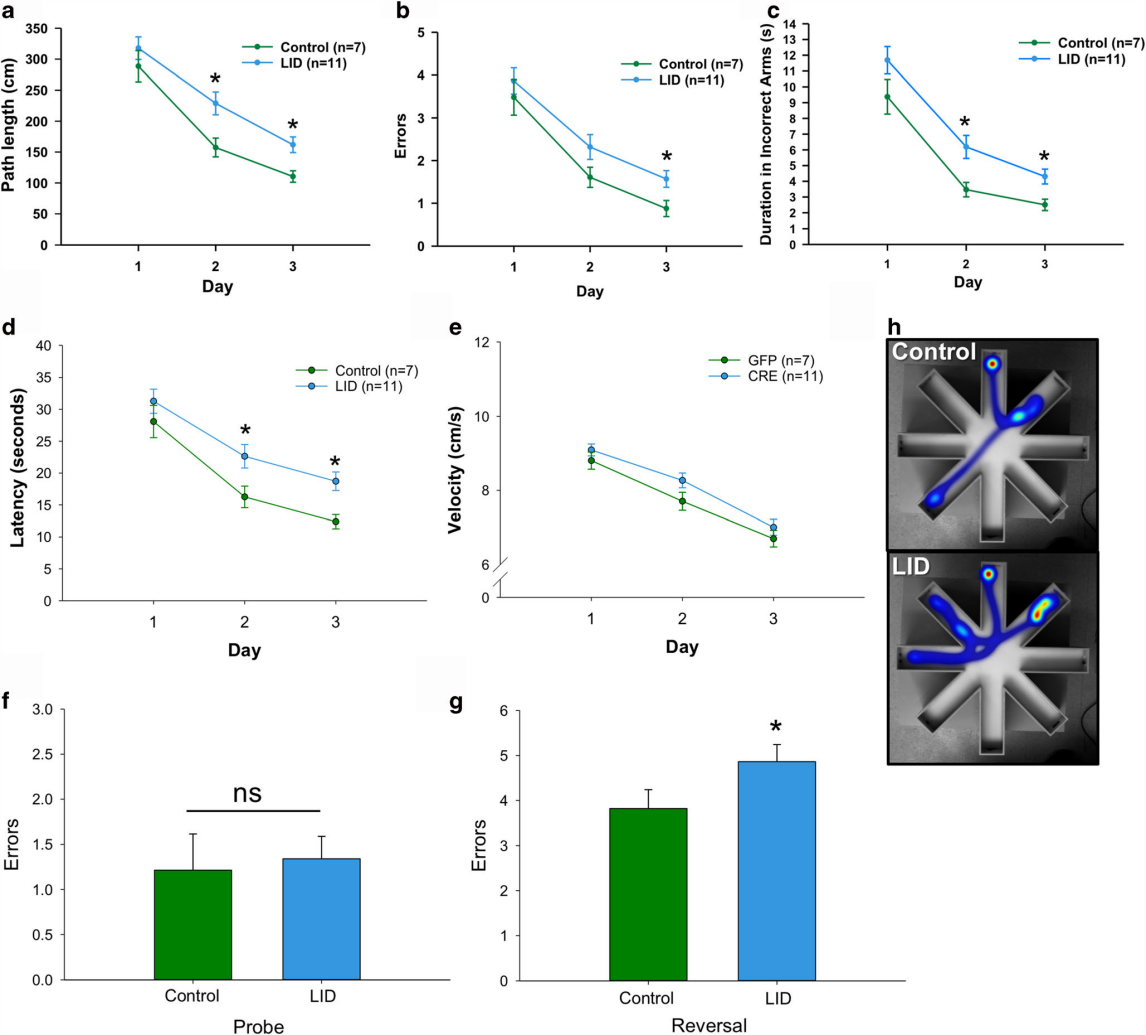
Circulating IGF-1 deficiency impairs hippocampal-dependent spatial acquisition and extinction. Mice (control and LID) were tested in the RAWM for spatial memory with 8 trials per day for 3 days (days 1–3 acquisition). One week post acquisition (day 10), mice were tested for memory of the learned target location (probe) followed by a reversal learning (extinction) of a new target location on day 11. During the acquisition phase, LID mice show increased path length to reach target (**a**), increased entries (**b**), and duration (**c**) into incorrect arms (errors) compared with controls. LID mice also show increased latency (**d**), albeit there were no differences in velocity between groups (**e**). **f** Memory of the learned target tested on day 10 was not different between groups. **g** LID mice made significantly more errors during reversal learning compared with controls. **h** Representative heat maps at the end of the third day of acquisition show that LID mice make more errors in spatial learning as measured by the aforementioned parameters

IGF-1 has pleiotropic effects on many tissues and cell types that vary between the sexes and tissue type [14, 16, 17]. Lifelong GH/IGF-1 deficiency has been shown to increase lifespan in many models including mice [59–63]. However, late life IGF-1 deficiency (relevant to humans) has only a modest effect on maximum lifespan in female but not in male mice, in part, by reducing cancer risk [14]. Numerous studies have shown that IGF-1 is neuroprotective and has pro-cognitive effects. To determine the impact of reduced serum IGF-1 on cognitive function, we tested control and LID mice in a radial arm water maze for hippocampal-dependent spatial learning and memory. Mice (18 months) were placed in the water maze and allowed to find a hidden platform and movement parameters were recorded (path length, etc.). LID mice took a longer path length (distance travelled; Fig. 4a) to find the platform over the 3-day period (acquisition) compared with controls (GFP). The slope of the lines also indicates that the LID mice are less efficient at acquiring the task compared with controls. LID mice also made more entries into incorrect arms (errors; Fig. 4b) and had a longer duration in incorrect arms (Fig. 4c) compared with controls. Time (s) to reach the target (latency) was also reduced in LID mice (Fig. 4d), while no differences were observed in the overall velocity to reach the target between the groups. Representative heat maps of mice (Fig. 4h; control and LID) illustrate the increased path length and errors taken to reach the target arm in LID mice compared with controls after the third day of training. Following acquisition, mice were tested for memory of the hidden platform on day 10. No differences were observed between the groups (Fig. 4f) in the animals’ ability to remember the location of the hidden platform (probe). On day 11, when the platform was switched to a new location, LID mice made more errors to reach the platform (Fig. 4g), suggesting that the LID mice are impaired in their ability to learn the new task.

### Decline in Circulating IGF-1 Decreases Brain Mitochondrial Efficiency and Increases Stress Response and Oxidative Damage

**Fig. 5:**
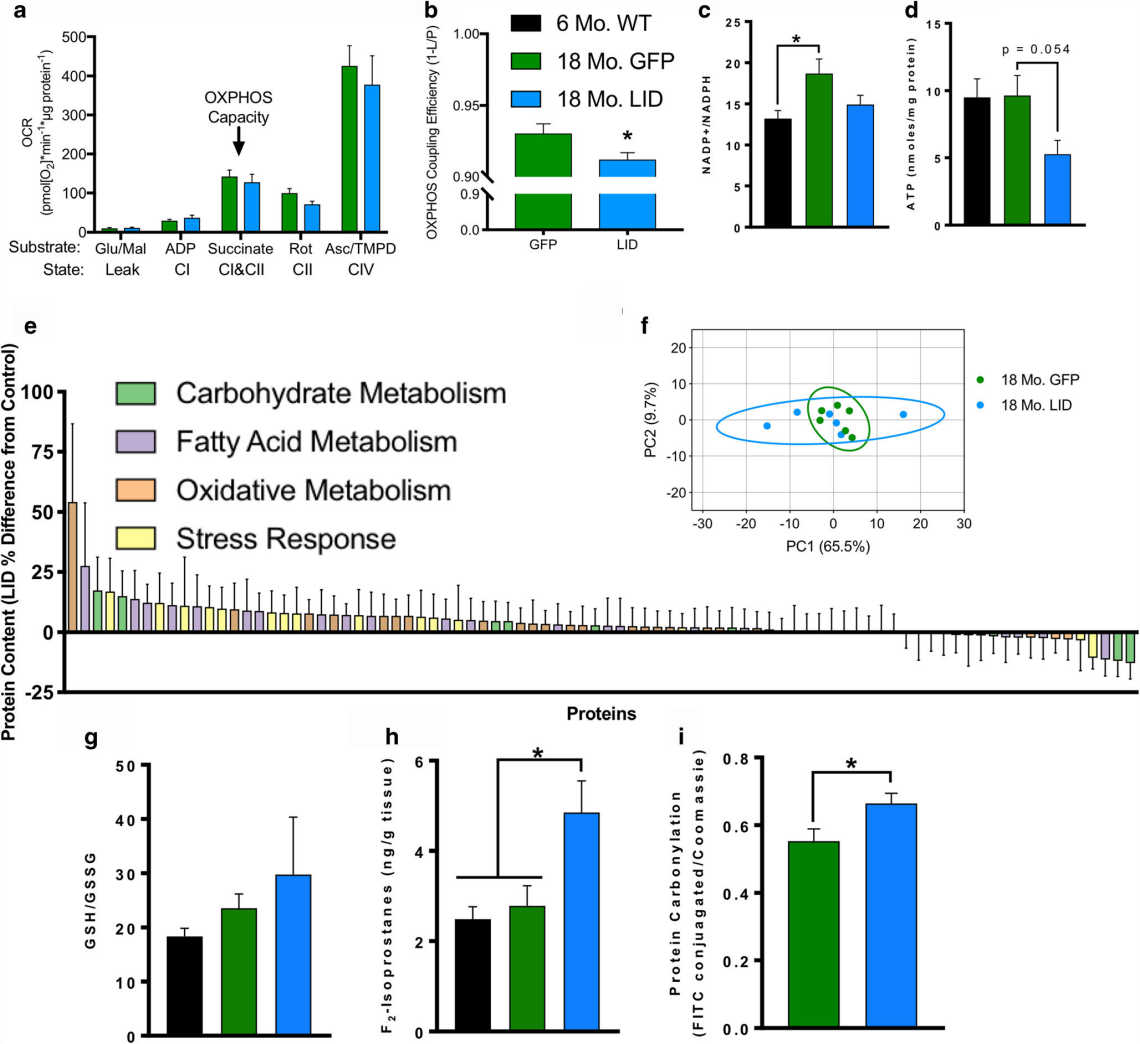
Reduction in circulating IGF-1 levels decreases brain mitochondrial efficiency and increases stress response and oxidative damage. **a** Oxygen consumption rate (OCR) and **b** OXPHOS coupling efficiency (1 – LEAK/OXPHOS Capacity) of hippocampus mitochondria isolated from 18-month GFP and LID mice measured in the O2K with Amplex UltraRed and normalized to μg mitochondrial protein (*n* = 8–10). **c** NADP+/NADPH ratio and **d** ATP concentration of cortex from 6-month WT and 18-month GFP and LID (*n* = 5–8). **e** Relative protein abundance and **f** principal component analysis (PCA) of metabolic and stress response proteins of isolated cortex protein from 18-month GFP and LID mouse brains by targeted mass spectrometry and selected reaction monitoring (*n* = 6). **g** Ratio of reduced (GSH) to oxidized (GSSG) glutathione and **h** level of F_2_-isoprostanes in the cortex of 6-month WT and 18-month GFP and LID mice (*n* = 5–8). **i** Protein carbonylation (FITC conjugated) normalized to Coomassie gel staining in the cortex of 18-month GFP and LID mice (*n* = 7–9). Statistical significance for experiments with two groups determined by two-tailed student’s *t* test (*p < 0.05), and statistical significance for experiments with three groups determined by ordinary one-way ANOVA with Tukey’s multiple comparison test (*p < 0.05). Box plots depicted as mean ± SEM. Six-month WT (black); 18-month GFP (green); 18-month LID (blue). Glutamate (Glu); malate (Mal); adenosine diphosphate (ADP); rotenone (Rot); ascorbate (Asc); N,N,N′,N′-tetramethyl-p-phenylenediamine dihydrochloride (TMPD); antimycin A (AmA); ETC complex I (CI); ETC complex II (CII); ETC complex IV (CIV)

IGF-1 has been shown to induce alterations in mitochondrial function [32] and protect against oxidative stress via sustained AKT activation [29, 30]. To assess mitochondrial function, we isolated mitochondria from 18-month GFP and LID hippocampi and measured oxygen consumption rate (OCR) and hydroperoxide production rate using O2K HRR. While we observed no significant difference in OCR, there is a modest increase in LEAK respiration and a decrease in OXPHOS capacity from isolated mitochondria from LID hippocampus resulting in decreased OXPHOS coupling efficiency (Fig. 5a, b). We observed no difference in hydroperoxide production rate, though there is a non-significant increase in hydroperoxide production capabilities with rotenone and antimycin A in LID hippocampus isolated mitochondria (Supplemental Figure 2A). After addition of rotenone, we observed a trend for an increase (*p* < 0.1) in hydroperoxide production as a percentage of oxygen consumed in 18-month LID cortex compared with GFP (Supplemental Figure 2B). We next asked how impaired hippocampus mitochondrial coupling efficiency would impact cellular energetics, so we measured concentrations of central energy metabolites in cortex of young and 18-month GFP and LID mice (Supplemental Table 1). Consistent with a decrease in mitochondrial coupling efficiency, we also observed a decrease in ATP concentrations in cortex from 18-month LID mice compared with GFP (Fig. 5d, Supplemental Table 1). However, no differences in NAD+, NADH, NADP+, NADPH, or the NAD+/NADH ratio were found (Supplemental Table 1). The NADP+/NADPH ratio was increased in 18-month GFP mice (Fig. 5c, Supplemental Table 1). Energy charge ratio, a measure of phosphorylated (ATP and ADP) versus total (ATP, ADP, AMP) adenosine, was also not significantly different (Supplemental Table 1) [33]. However, the decrease in mitochondrial coupling efficiency and ATP content in 18-month LID mice suggested a decrease in energetic capability in IGF-1-deficient brains.

Protein from hippocampi of 18-month GFP and LID mice was isolated and targeted proteomics analysis was performed to measure how impaired energy production affects protein content of central metabolic and stress response pathways (Supplemental Table 2). No individual protein measured passed our false discovery rate (FDR) threshold (*p* < 0.05) (Fig. 5e). Of the proteins measured, most were increased in expression in LID mice (69 increased versus 17 decreased) and higher variability was seen in principal component analysis (PCA) of LID compared with GFP mice (Fig. 5e, f). Increased variability in LID mice protein is consistent with the concept of increased age-related heterogeneity [64].

Finally, oxidative stress was assessed in the cortex using the glutathione ratio, lipid peroxides, and protein carbonyls. We found no differences in the individual concentrations of reduced (GSH) or oxidized glutathione (GSSG), but we did observe a non-significant increase in the GSH/GSSG ratio in 18-month LID mice as well as a statistically significant increase in variability (Bartlett’s test *p* < 0.0001) (Fig. 5g, Supplemental Table 1). We observed a significant increase in F_2_-isoprostane concentration in cortex from aged LID mice compared with young and old controls (Fig. 5h). Previous reports indicate that oxidative damage to proteins can cause the formation of protein carbonyls [65], and we found a significant increase in protein carbonyls in cortex from 18-month LID mice compared with GFP mice (Fig. 5i, Supplemental Figure 2A, B).

## Discussion

The highly conserved insulin/IGF pathway has been extensively studied [14, 66–72] in the context of aging and neurodegeneration [11, 73]. While early evidence suggested that lower levels of IGF-1 were beneficial for lifespan, recent extensive studies indicate that these findings were limited to *C. elegans* and drosophila that exhibit common insulin/IGF-1 receptor pathways [15]. In mammals, the effects of IGF-1 are both tissue and sex specific [11, 14–17, 74]. Furthermore, prevailing evidence suggests that age-related deficiency in IGF-1 is associated with cognitive impairment, vascular rarefaction [70], and diminished clearance of amyloid β (Aβ) [75–78]. We have previously shown that reduced IGF-1 signaling in astrocytes impairs mitochondrial metabolism and working memory [32]. In light of the controversy of the central and peripheral effects of IGF-1, we investigated whether these effects are mediated through the modulation of mitochondrial function. In this study, we show that circulating IGF-1 deficiency has varied effects on peripheral and brain energy metabolism that manifests as a decline in cognitive function but in contrast has marginal effects on peripheral mitochondrial function.

Loss of muscle mass/sarcopenia is a primary component of frailty and morbidity in aged individuals. Both insulin and IGF-1 are potent regulators of protein synthesis, muscle metabolism, and growth through shared downstream signaling pathways [79–86]. Our data showed no differences in muscle mitochondrial oxygen consumption rate in LID mice at 24 months of age compared with age-matched controls, although muscle mass and OXPHOS capacity declined with age as expected. Consistent with previous reports [34], hydroperoxide production was unaffected with age or with reduced circulating IGF-1. These data suggest that reduced circulating IGF-1 levels did not alter the age-related decline in muscle mass. However, these effects may need to be functionally characterized in these mice using exercise/stimulation of skeletal muscle to detect capacity for hypertrophy since muscle-specific overexpression of IGF-1 has been shown to prevent age-related atrophy and myopathies [44, 87]. Surprisingly, we found a decrease in hydroperoxides produced by aged GFP and LID mice. However, when hydroperoxide production is represented as a proportion of oxygen consumed, we find no difference with age. This suggests that the age-related decrease in hydroperoxides is related to the decline in oxygen consumption rates and is not caused by an increase or decrease in rates of electrons forming reactive oxygen species from the electron transport chain (ETC). Furthermore, when we added electron transport chain inhibitors, we saw no difference in capacity to produce hydroperoxides with age, which was recently reported in human muscle biopsies [88].

Growth hormone levels are reported to increase both fat metabolism and insulin resistance [47–49, 89, 90]. We have previously reported that circulating IGF-1 deficiency results in a compensatory rise in GH levels [14]. In accordance with these reports, LID mice are insulin resistant, display increased fat metabolism, and have reduced fat mass. While GH has direct lipolytic effects on mature adipocytes, IGF-1 administration in GH-deficient human subjects synergistically increased lipid oxidation in combination with GH supplementation [91]. Thus, reduced circulating IGF-1 levels may augment the effects of GH on fat mobilization. While muscle respiration depends on electrons entering the electron transport chain from both complexes I and II, fat mitochondria depend primarily on complex II-linked respiration. ETC complex II is highly sensitive to oxidative damage, which could partially explain the age-related decrease in OXPHOS capacity [92]. Basal hydroperoxide production was approximately 4.5 times higher in fat biopsies than in muscle. However, because this rate did not increase with addition of electron transport chain inhibitors, it is likely that the source of the hydroperoxides is not the electron transport chain. These basal hydroperoxides interacting with Amplex UltraRed could be hydrogen peroxide derived from NAD(P)H oxidases via production of superoxide anions or by reaction with lipid peroxides [36, 93].

The effects of GH/IGF-1 on lifespan/health span have remained controversial especially related to peripheral and central tissue functions. Mouse models of early life GH/IGF-1 deficiency are reported to have beneficial effects on health span and lifespan [94, 95]. However, the confounding effects of altered development and compensatory mechanisms that promote survival in the early life GH/IGF-1-deficient models impair interpretation and their significance for aging/health span effects. Adult-onset IGF-1/IGFR deficiency have minimal effects on lifespan in males and increases lifespan in females [14, 96]. We previously reported that the adult-onset liver-IGF-1-deficient (LID) mice displayed a significant decline in spatial learning assessed by the Barnes maze [14]. Our current data support these previous findings in that LID mice have significant impairments in spatial learning and reversal learning, but recall of a learned memory is not affected. These data suggest that initial processing by the hippocampus in consolidation is sensitive to reduced circulating IGF-1 levels. Reversal learning is the animal’s ability to forget the previous location and learn the platform location. Reversal learning in mice has been evaluated in a variety of behavioral tasks, including spatial learning with Morris water maze, T-maze [97], and eight arm maze [98]. In our study, LID mice exhibited a deficit in reversal learning that is indicative of a dysfunction in the coordinated actions of these brain regions associated with reversal learning such as the orbitofrontal cortex, dorsal striatum, and amygdala [99–103].

These data are consistent with reports that have shown beneficial cognitive effects from GH/IGF-1 replacement [10, 54, 66, 73, 104, 105]. Additionally, GH-deficient mice have reduced hippocampal ATP levels, which is improved upon GH/IGF-1 replacement [31]. More recently, overexpression of IGF-1 centrally has been shown to protect against age-related cognitive dysfunction in male mice [106]. Interestingly, central IGF-1 administration has also been shown to improve peripheral insulin action [107]. Low doses of IGF-1 have also shown to improve liver mitochondrial function in aging rats [53]. Our data show reduced hippocampal OXPHOS coupling efficiency and brain ATP levels in LID mice with a concomitant increase in oxidative damage in the brain that may underlie the cognitive deficits in this model. These data provide further support for the concept that IGF-1 has beneficial effects in the brain with varied peripheral effects. Future studies examining mitochondrial metabolism in various brain regions of IGF-1-deficient mice will be important to understand the role of regional differences in energy metabolism on cognitive function.

In conclusion, we have shown that the effects of IGF-1 are multifaceted. Certainly, much of the evidence in the literature, which is supported by our own findings, suggests that IGF-1 is protective for cognitive function, and that there exists an apparent dichotomy between the systemic and central regulation of IGF-1 actions. Thus, the evolutionary advantage of reduced peripheral IGF-1 and increased lifespan with respect to reduced cancer incidence and progression may be offset by a central deficit that serves minimal evolutionary impact. Furthermore, IGF-1 effects on energy metabolism appear to underlie, in part, the differences between the central and peripheral effects of IGF-1. Therefore, resolution of the controversies underlying IGF-1 signaling between invertebrate and mammalian models can be achieved only through rigorous investigation of downstream signaling pathways in a tissue/cell-specific manner.

## Electronic supplementary material

ESM 1 (DOCX 13 kb)

ESM 2 (TIF 24676 kb)

ESM 3 (TIF 24677 kb)

ESM 4 (TIF 24677 kb)

ESM 5 (PNG 68 kb)

High Resolution (TIF 24678 kb)

ESM 6 (PNG 660 kb)

High Resolution (TIF 24675 kb)
