# Supplementary material for: Disparate Central and Peripheral Effects of Circulating IGF-1 Deficiency on Tissue Mitochondrial Function
Source: Mol Neurobiol. 2019 Nov 15;57(3):1317–31. doi: 10.1007/s12035-019-01821-4 (PMC7060968; doi:10.1007/s12035-019-01821-4)
Supplement: Supplementary file 1 — (DOCX 13 kb) [file 12035_2019_1821_MOESM1_ESM.docx]

**Supplemental methods**

***Targeted Quantitative Proteomics***

100µg total protein of each samples was taken, mixed with SDS to a final concentration of 1%, and 8pmol BSA added as an internal standard. The proteins were precipitated with acetone. The dried protein pellet was reconstituted in 100µL Laemmli sample buffer and 20µL run into as short (1.5cm) SDS-PAGE gel. The gels were fixed and stained. Scans of the stained gels are shown in Figure 1. Each sample was cut from the gel as the entire lane and divided into smaller pieces. The gel pieces were washed to remove the Coomassie blue then reduced, alkylated, and digested overnight with trypsin. The mixture of peptides was extracted from the gel, evaporated to dryness in a SpeedVac and reconstituted in 150µL 1% acetic acid for analysis. The analyses were carried out on our TSQ Vantage triple quadrupole mass spectrometry system. The HPLC was an Eksigent splitless nanoflow system with a 10cm x 75µm i.d. C18 reversed phase capillary column. 7µL aliquots were injected and the peptide eluted with a 60min gradient of acetonitrile in 0.1% formic acid. The mass spectrometer was operated in the selected reaction monitoring mode. For each protein, the method was developed to measure 2 ideal peptides. Assay for multiple proteins were bundled together in larger panels. Data were analyzed using the program SkyLine to determine the integrated peak area of the appropriate chromatographic peaks. The response for each protein was calculated as the geometric mean of the two peptide area. These values were normalized to the response for the BSA standard. Multiple housekeeping proteins were also monitored.

**Supplemental Figure Legends**

**Supplemental Figure 1. Hydroperoxide production in isolated hippocampus mitochondria (A)** Hydroperoxide production rate (ROS) production in hippocampus mitochondria isolated from 18-month GFP (green) and LID (blue) mice measured in the OROBOROS O2k with Amplex UltraRed and normalized to µg mitochondrial protein (n=8-10). **(B)** Isolated hippocampus mitochondria Amplex UltraRed Reaction Rate (ROS) as a percentage of oxygen consumption rate (OCR) (n=8-10). Statistical significance determined by two-tailed student’s t-test (*p < 0.05). Box plots depicted as mean ± SEM. Glutamate (Glu); Malate (Mal); Adenosine diphosphate (ADP); Rotenone (Rot); Ascorbate (Asc), Antimycin A (AmA).

**Supplemental Figure 2. Protein Carbonylation in Cortex.** Protein carbonylation detected using **(A)** avidin‐fluorescein isothiocyanate (FITC) affinity staining and **(B)** coomassie gel staining in cortex from 18-month GFP and LID mice (n=7-9). *p<0.05

**Supplemental Table 1. Energy Charge and Glutathione in Cortex and eWAT.** Analyzed from cortex of 6-month WT and 18-month GFP and LID mice (n=5-8) and eWAT of 6-month WT and 24-month GFP and LID mice (n=6). Mean ± standard deviation. Statistical significance determined by ordinary one-way ANOVA with Tukey’s Multiple Comparison Test (*p < 0.05 compared to 6-month control).

**Supplemental Table 2. Individual Protein Content from Targeted Proteomics in Cortex.** Protein content of individual proteins analyzed from cortex of 18-month GFP and LID mice (n=6). Mean ± standard deviation.
