# Supplementary figures and images for: Disparate Central and Peripheral Effects of Circulating IGF-1 Deficiency on Tissue Mitochondrial Function
Source: Mol Neurobiol. 2019 Nov 15;57(3):1317–31. doi: 10.1007/s12035-019-01821-4 (PMC7060968; doi:10.1007/s12035-019-01821-4)

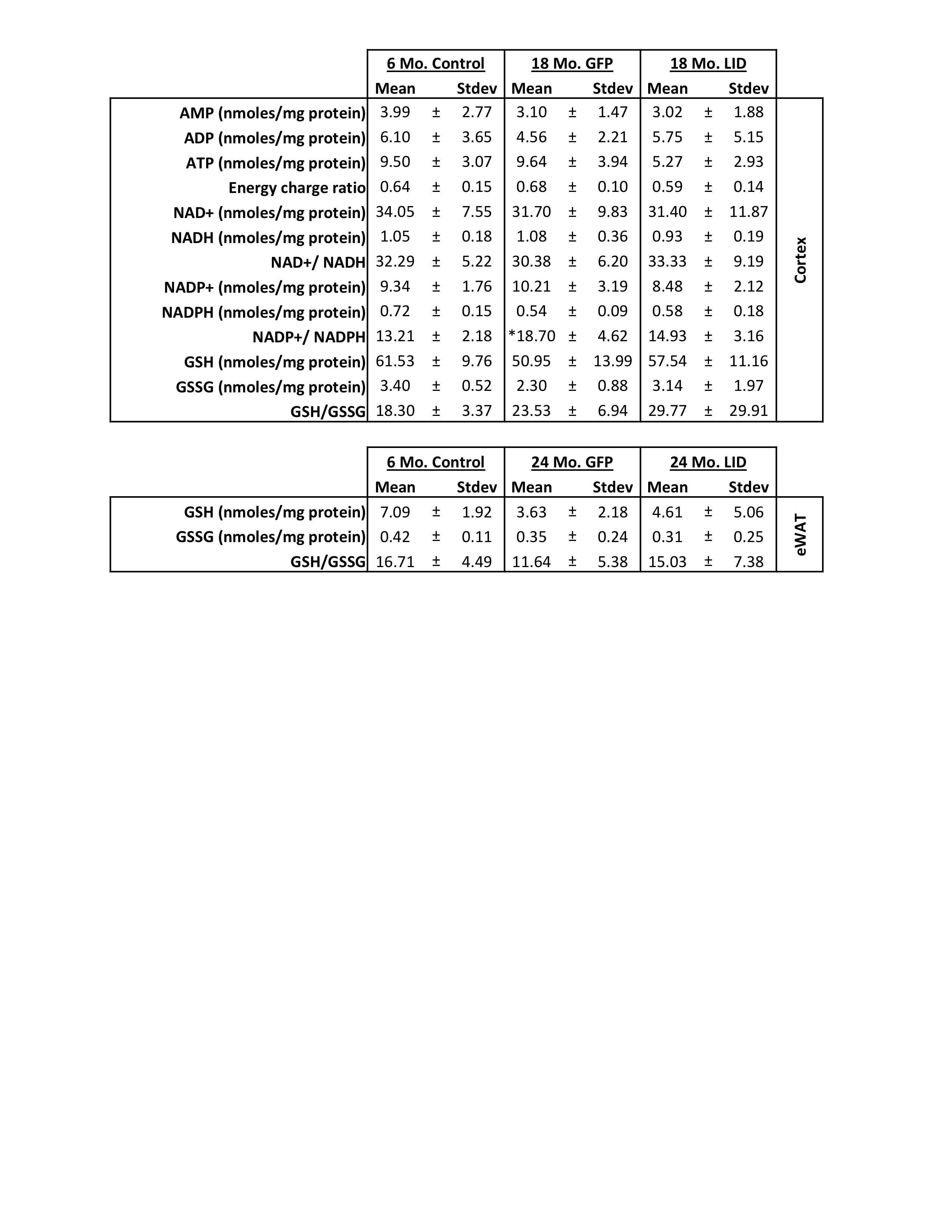

Supplement: Supplementary file 2 — (TIF 24676 kb) [file 12035_2019_1821_MOESM2_ESM.tif]

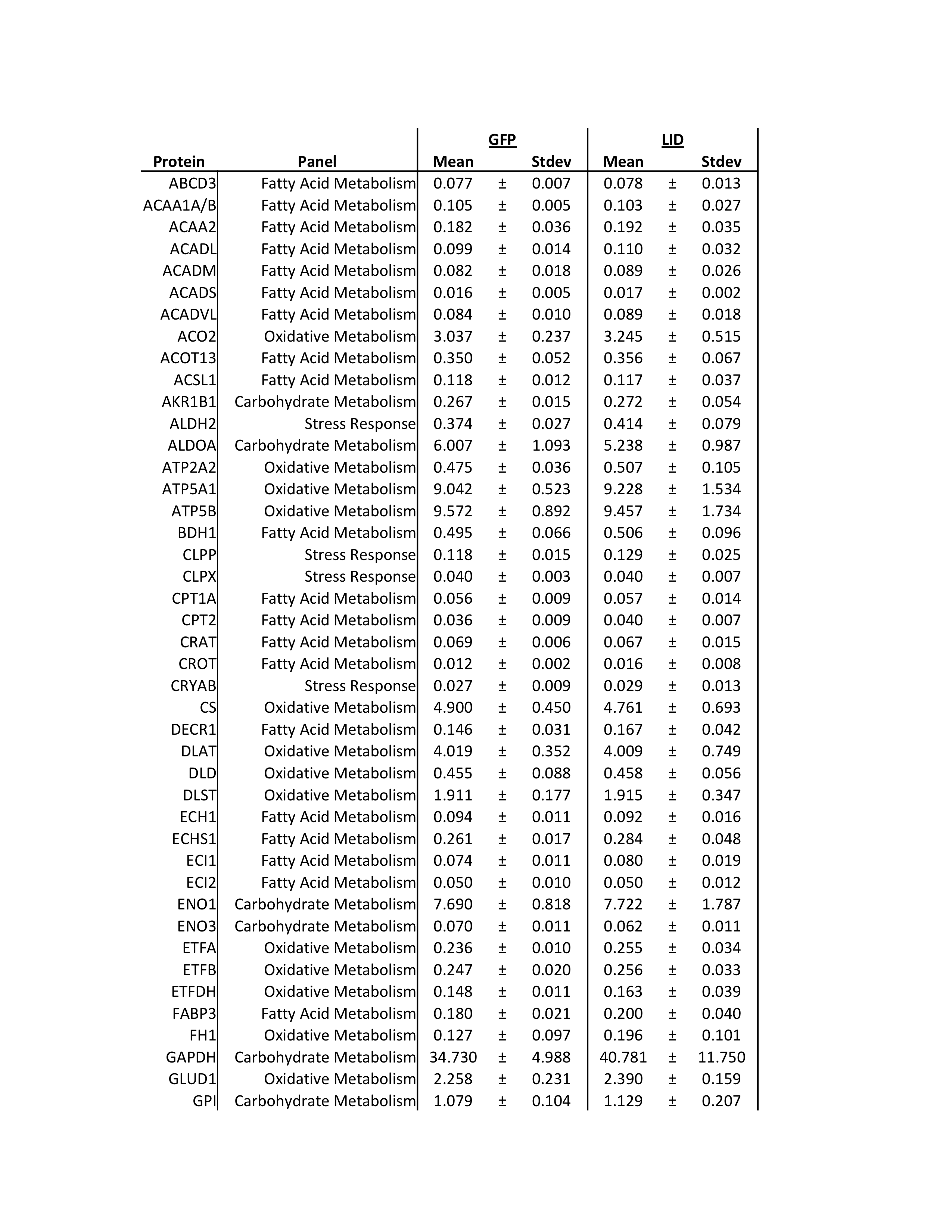

Supplement: Supplementary file 3 — (TIF 24677 kb) [file 12035_2019_1821_MOESM3_ESM.tif]

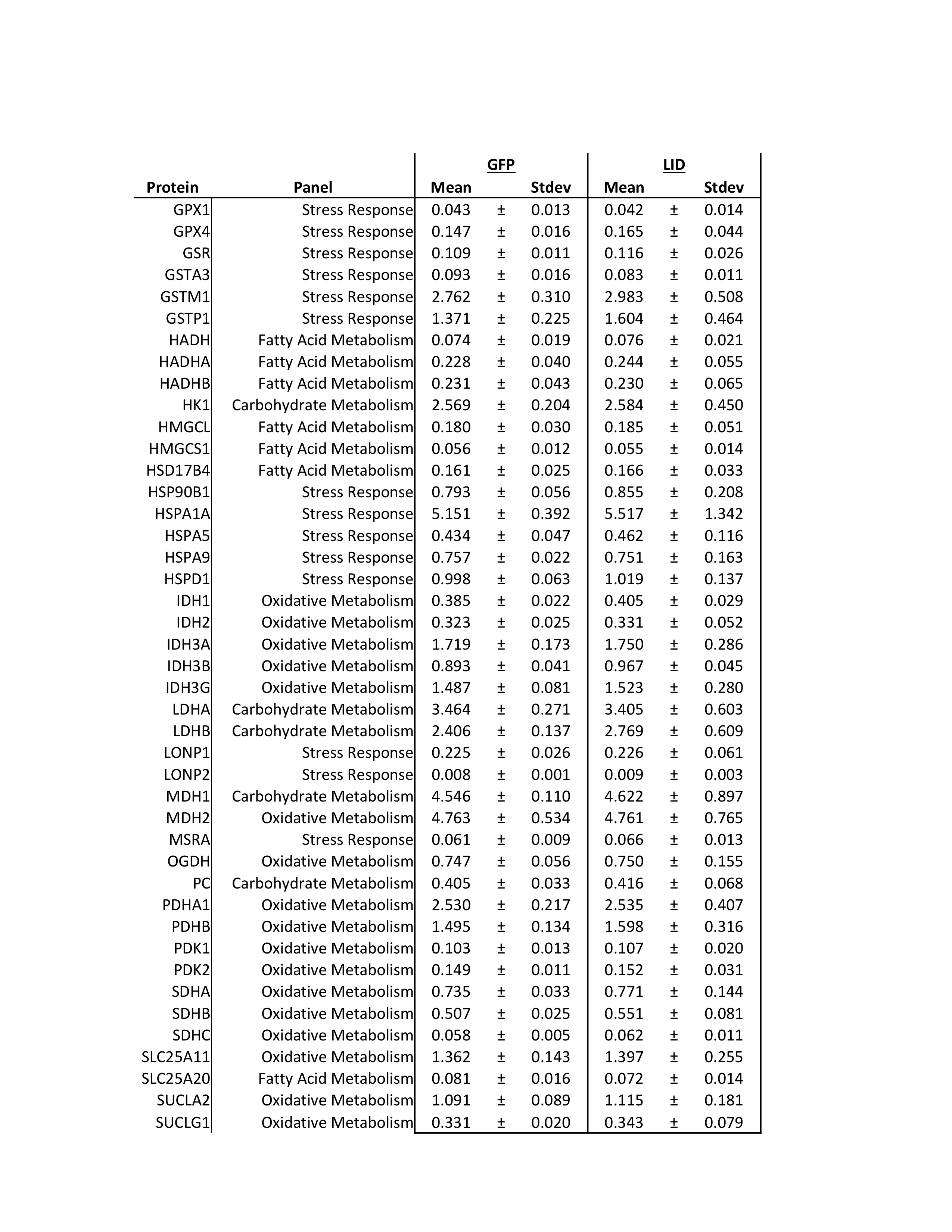

Supplement: Supplementary file 4 — (TIF 24677 kb) [file 12035_2019_1821_MOESM4_ESM.tif]

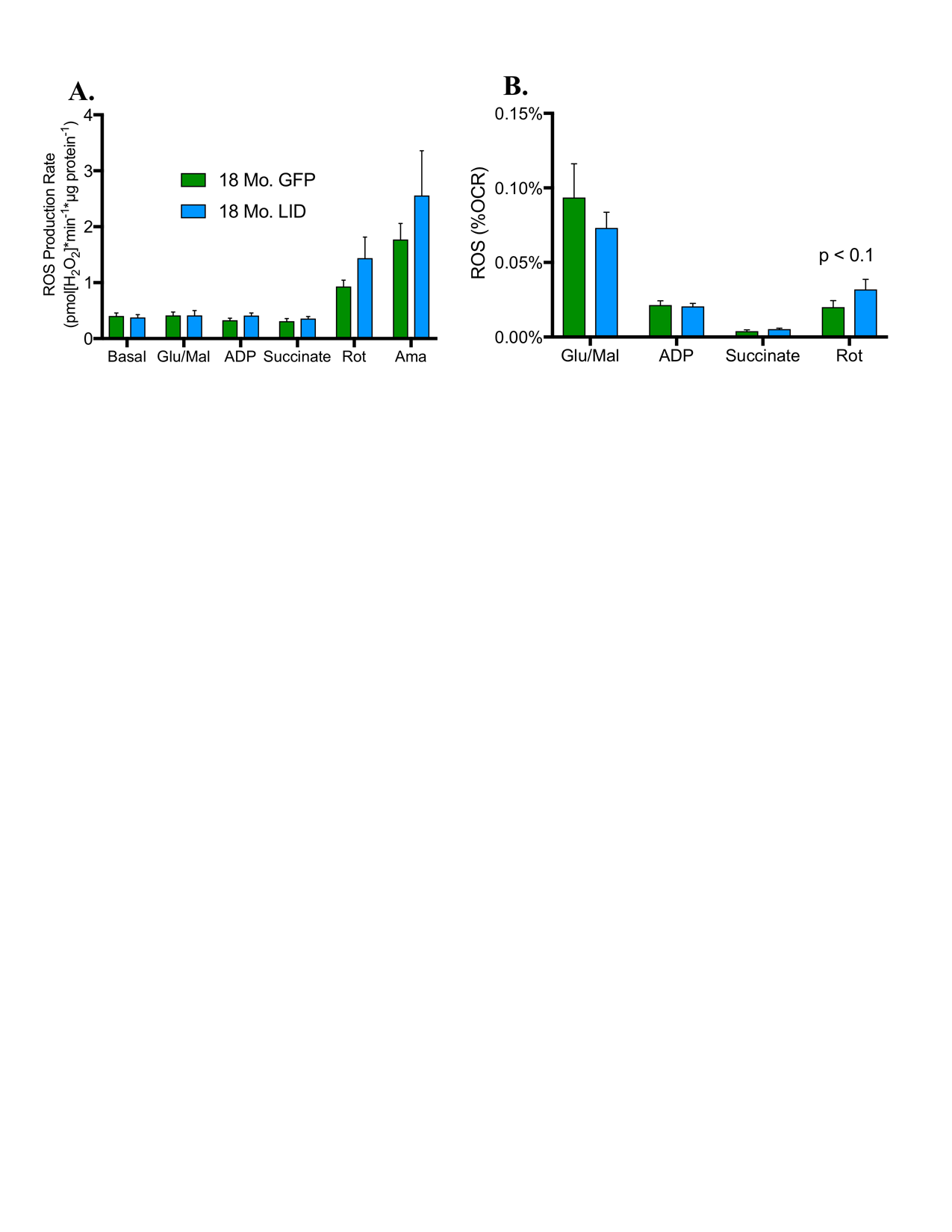

Supplement: Supplementary file 5 — (PNG 68 kb) [file 12035_2019_1821_Fig6_ESM.png]

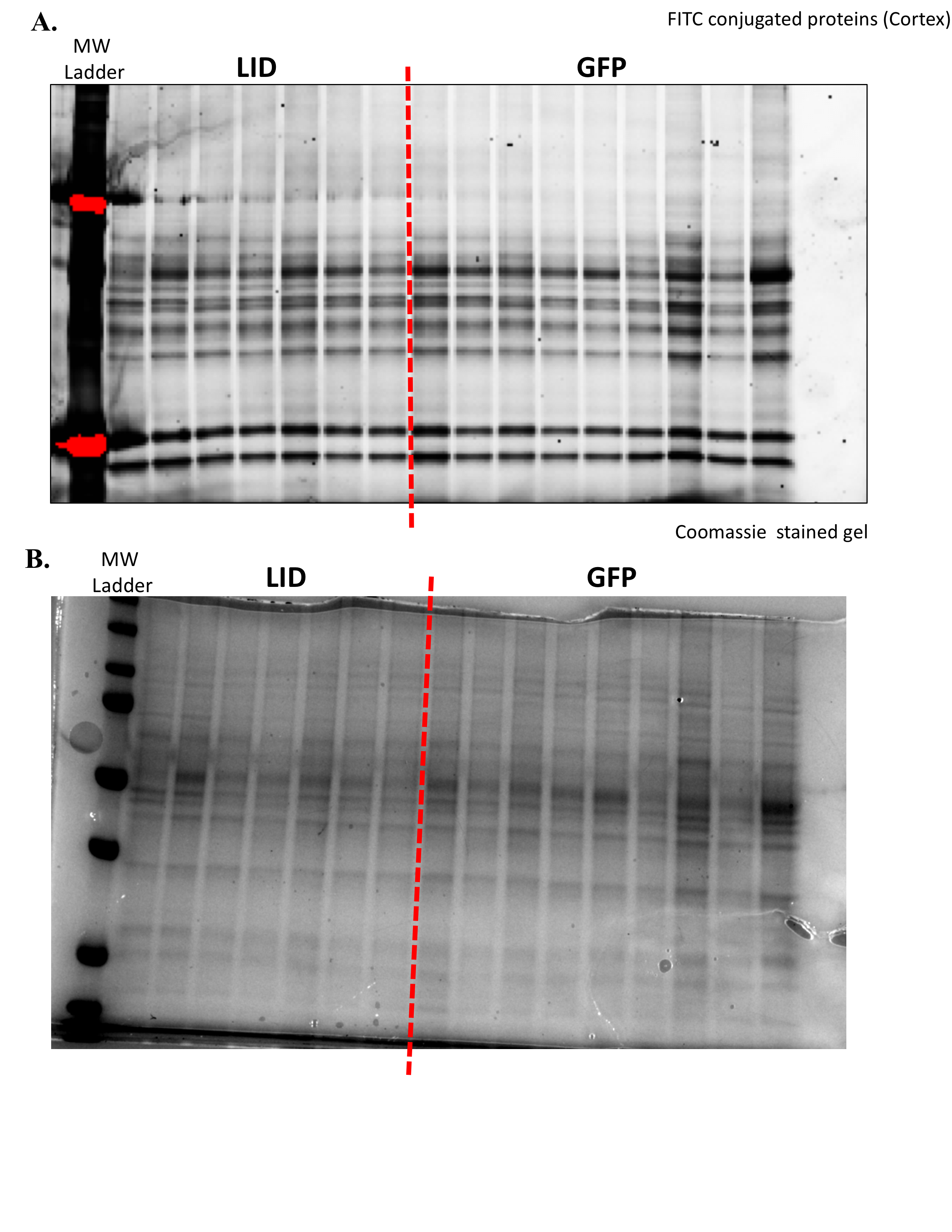

Supplement: Supplementary file 6 — High Resolution (TIF 24678 kb) [file 12035_2019_1821_MOESM5_ESM.tif]

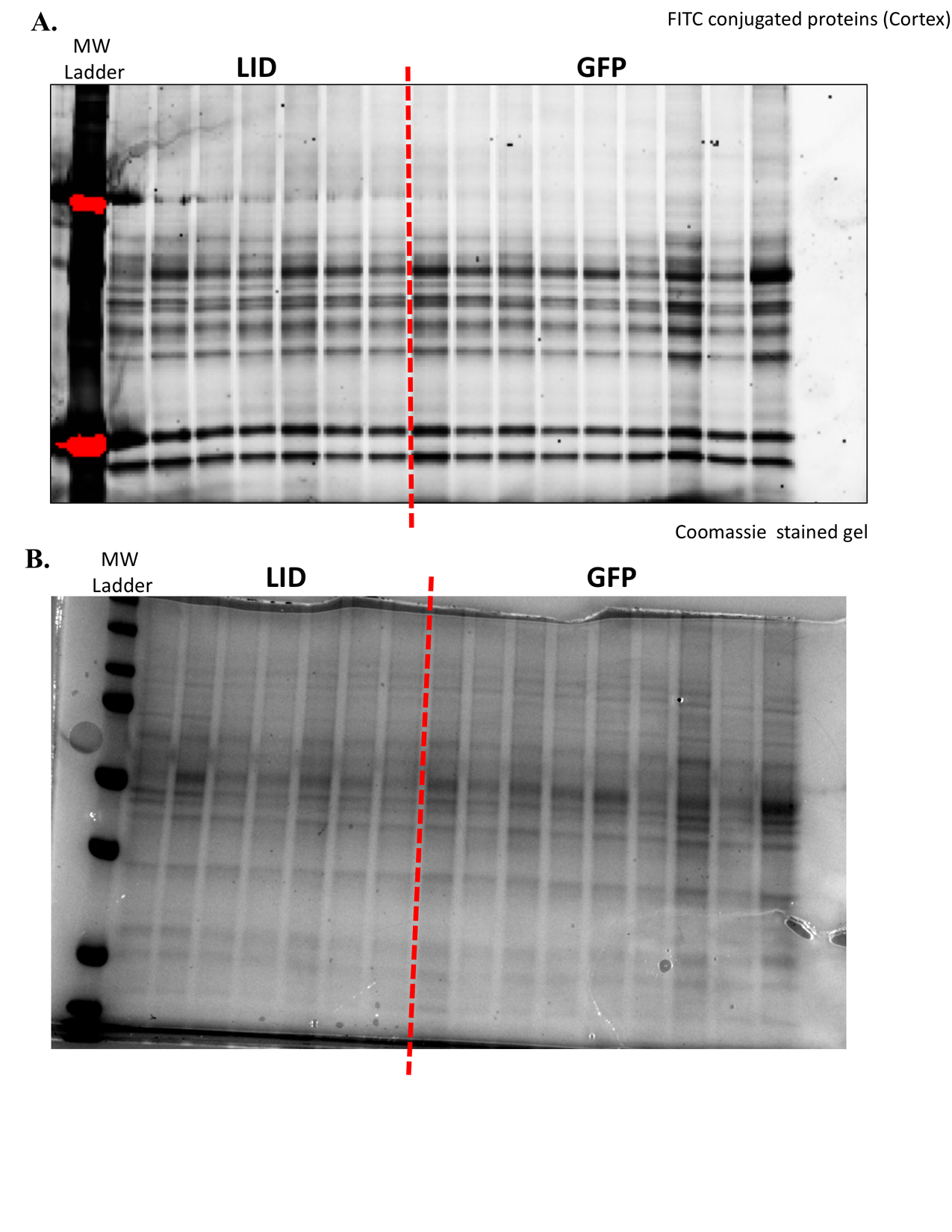

Supplement: Supplementary file 7 — (PNG 660 kb) [file 12035_2019_1821_Fig7_ESM.png]

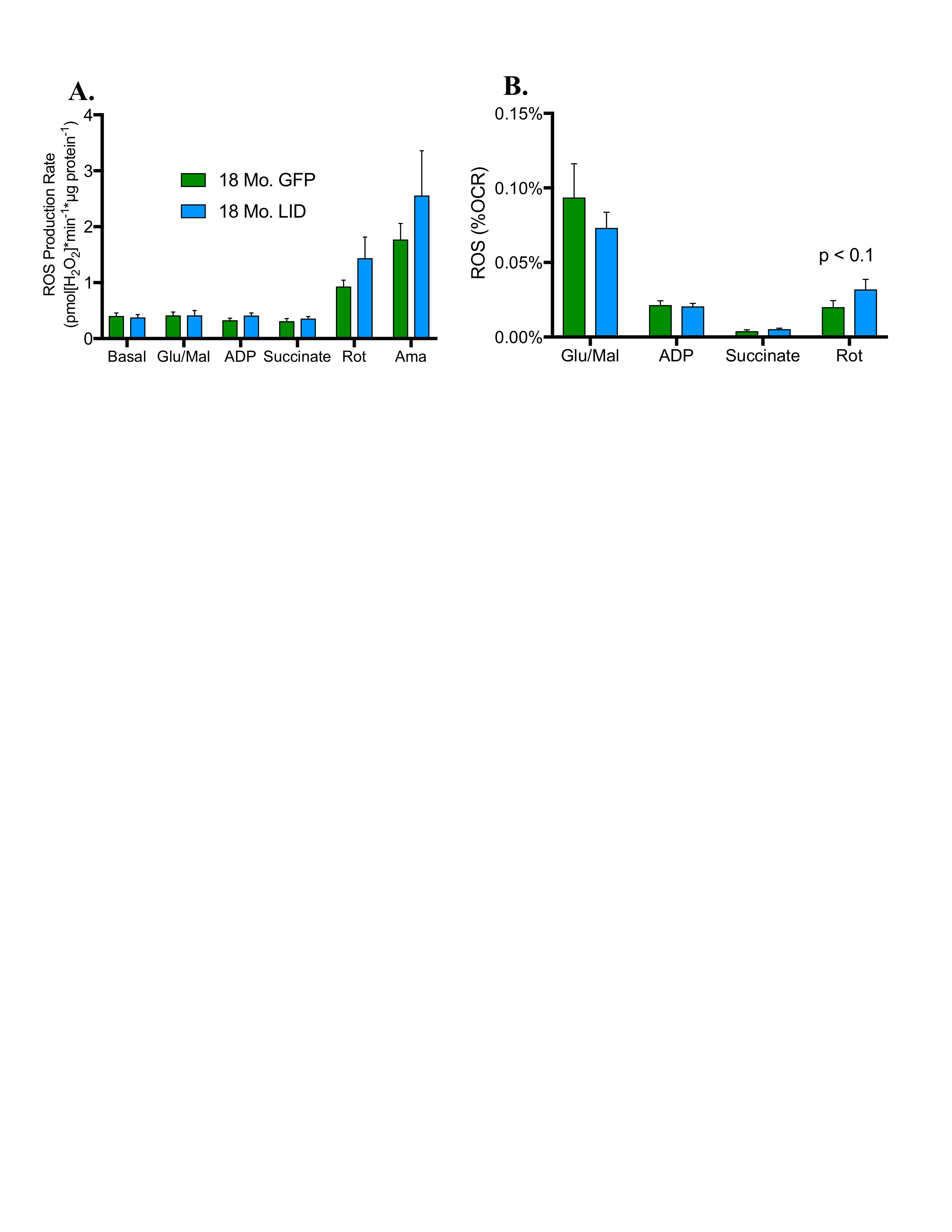

Supplement: Supplementary file 8 — High Resolution (TIF 24675 kb) [file 12035_2019_1821_MOESM6_ESM.tif]
